# Action of Chloroform

**Published:** 1882-06

**Authors:** 


### ACTION OF CHLOROFORM.

In the discussion which has for several weeks occupied the attention of the Académie de Medécine, regarding the symptoms observed during chloroformization, M. Vulpian, at a recent séance (March 28th), expressed the following opinions concerning the practical and experimental questions brought forward by the different speakers :

The symptoms induced by chloroform are observed generally at three principal periods—either after the first few inspirations, or while the patient is gradually coming under the influence of the drug, or, again, within a few hours or even a few days after chloroform has been administered.

Excitation of the superior ends of the upper laryngeal nerves induces arrest of respiration, and the same effect is obtained when one of the nerves controlling the upper segments of the respiratory organs is touched with a camel's hair brush pencil wet with chloroform. During his experiments M. Vulpian remarked that animals, dogs especially, do not support as well as man the action of chloroform. The symptoms observed are due to arrest of the heart (cardiac syncope) or to that of the respiration (respiratory syncope). Chloroform does not invade successively the different organs ; it acts immediately and at once on all the tissues. The respiratory centre seems to offer a notable resistance to its action, but is not entirely spared.

If the pneumogastric nerve be divided in an animal under the influence of chloral or chloroform, the animal continues to breathe, notwithstanding the section of the nerve. Excitation of the central end determines sudden and immediate arrest of the respiration, lasting from one-half to one minute. Excitation of the peripheric end in a chloralized animal produces complete and definite arrest of respiration, and while in the non-chloralized animal there is but a momentary arrest of the heart, in the other the heart beat ceases and the animal succumbs. Anæsthesia acts, then, on the cells of the respiratory centres, also on those of the sympathetic ganglia, which control the action of the heart, and these ganglia are no longer capable of resuming their functions when these have been once abolished by any excitation, electric or otherwise.

In unfortunate cases, when all the organs are under the influence of chloroform, the animal continues to breathe and the heart does not cease beating ; but if too large a dose be administered, or any excitation of the pneumogastric take place, then respiratory or cardiac dyspnœa supervenes and the animal succumbs. Symptoms may supervene while operations are being performed, when anæsthesia is complete, demonstrating that the cord conserves its powers of conduction, even when under the influence of chloroform ; and that peripheric excitations are transmitted without cessation to the bulb. Under such conditions syncope, of bulbar origin frequently supervenes.

On animals cardiac syncope is of greater gravity than that induced by arrest of respiration, for in the latter case artificial respiration may and should be resorted to. M. Vulpian concluded by saying that chloroformization is never exempt from danger, as syncope is always imminent. —*Medical and Surgical Report.*

